# Minimal Clinically Important Difference in Patients with Knee Cartilage Lesions Treated with a Cell-Free Scaffold Implantation

**DOI:** 10.1177/19476035251322730

**Published:** 2025-04-15

**Authors:** Luca De Marziani, Angelo Boffa, Marco Franceschini, Luca Andriolo, Alessandro Di Martino, Stefano Zaffagnini, Giuseppe Filardo

**Affiliations:** 1 Clinica Ortopedica e Traumatologica 2, IRCCS Istituto Ortopedico Rizzoli, Bologna, Italy; 2 Applied and Translational Research (ATR) Center, IRCCS Istituto Ortopedico Rizzoli, Bologna, Italy; 3 Faculty of Biomedical Sciences, Università della Svizzera Italiana, Lugano, Switzerland

**Keywords:** MCID, knee, cartilage, osteochondral, scaffold

## Abstract

**Objective:**

The aim of this study was to establish the minimal clinically important difference (MCID) thresholds for the International Knee Documentation Committee (IKDC) subjective and Visual Analogue Scale (VAS) pain scores in patients affected by knee chondral and osteochondral lesions treated with cell-free scaffold implantation.

**Design:**

For the MCID definition, 186 patients who underwent an osteochondral scaffold implantation were included. Patients were evaluated through the IKDC subjective and VAS pain scores at baseline, 12 and 24 months. The MCID was calculated using a distribution-based method for both IKDC subjective and VAS pain scores at 12 and 24 months, as well as with an anchor-based method.

**Results:**

The MCID values were 10.1 and 1.5 for the IKDC subjective and VAS pain scores, respectively, both at 12 and 24 months of follow-up. The rate of patients who achieved the MCID was 83% at 12 months and 88% at 24 months. The anchor-based method led to higher MCID values. Factors identified to increase the probability to reach the MCID were younger age (*P* = 0.042), male sex (*P* = 0.042), and lateral femoral condyle lesions (*P* = 0.002), while patellar lesions were less likely to reach the MCID (*P* = 0.009).

**Conclusions:**

This study defined the MCID values for the IKDC subjective and VAS pain scores after treatment with a cell-free biomimetic scaffold, with 88% of the patients achieving clinically relevant results at 2 years. Younger patients, males and lateral femoral condyle lesions were more likely to reach the MCID. However, the identified thresholds can be influenced by the method chosen, which warrants caution when interpreting study results.

## Introduction

Knee cartilage lesions can be a debilitating pathology with a high prevalence, being present in more than 60% of patients undergoing knee arthroscopy.^1,2^ These lesions present a limited self-healing capacity and, if untreated, they can easily degenerate leading to the early development of osteoarthritis.^3,4^ Thus, surgical procedures represent an important solution to provide clinical improvement and possibly delay joint degeneration.^5^ Several surgical treatments have been proposed to address lesions of the articular surface, from reparative to regenerative techniques.^6,7^ Among them, increasing evidence is supporting the use of innovative cell-free biomimetic scaffolds, which aim to provide a biodegradable three-dimensional structure that favors chondral or even osteochondral tissue regeneration.^8,9^ These scaffolds have been explored in several studies, reporting a statistically significant clinical improvement in patient-reported outcome measures (PROMs), including both pain with the Visual Analogue Scale (VAS) and functional evaluation, such as the International Knee Documentation Committee (IKDC) score.^10^ However, a statistical improvement does not always reflect a clinical improvement, which makes it difficult to understand the real clinical relevance of these findings.^11^

To this regard, the minimal clinically important difference (MCID) is a psychometric measure that has been developed to assist clinical researchers in interpreting PROMs.^12^ It is defined as the smallest difference in a specific PROM score that patients perceive as beneficial, referring to the amount of absolute change in a PROM score that relates to a clinical improvement.^13^ Recently, the use of MCID for specific PROMs has become increasingly widespread in clinical research to better understand the clinical efficacy of a given treatment, to properly interpret the results from trials, and to define clinical practice guidelines.^14,15^ However, MCID thresholds can vary based on the type of treatment, thus requiring the definition and application of specific MCIDs for each specific treatment.^16,17^ MCID values for the IKDC and the VAS have been calculated in patients undergoing different cartilage procedures, including microfracture technique, autologous chondrocyte implantation, and osteochondral allografts,^11,18-20^ while the MCID threshold remains to be defined to assess the clinical relevance of cell-free scaffold results in the treatment of articular surface lesions.

The aim of this study was to establish the MCID thresholds for the IKDC subjective and VAS pain scores in patients affected by knee chondral and osteochondral lesions treated with cell-free scaffold implantation.

## Methods

The present study analyzed prospectively collected PROMs (IKDC subjective score and VAS pain) from a database of patients with knee chondral and osteochondral lesions treated with a cell-free biomimetic scaffold from January 2007 to January 2022. Ethic Committee approval was obtained for the study protocol (Prot. No. 0020747) and informed consent was obtained at the time of patient enrollment. Cell-free scaffold implantation was indicated in patients with clinical symptoms, such as knee pain or swelling and with International Cartilage Repair Society (ICRS) grade 3-4 chondral and osteochondral lesions or osteochondritis dissecans (OCD) involving the femoral condyles, trochlea, patella, or tibial plateau. The procedures were performed in a specialized orthopedic clinic by a surgical team expert in the treatment of cartilage lesions. Exclusion criteria were untreated malalignment or ligament instability or other general medical conditions at the time of surgery, including infections, neoplastic, metabolic, and inflammatory pathologies. Patients with axial malalignment or ligament instability underwent a realignment procedure or ligament reconstruction during the same surgical session of the scaffold implantation.

Baseline demographic and clinical patients’ characteristics were collected from all patients to investigate their influence on the results in terms of clinical relevance: 186 patients were included based on the presence of the specific data requested for quantification of the MCID, including a specific anchor question and the scores at both the 12- and 24-month follow-ups. Demographic and clinical characteristics are reported in detail in Table 1.

**Table 1.** Included Patients’ Characteristics.

Sex, M/W	139/47
Age, years	31.8 ± 11.1
BMI, kg/m^2^	24.0 ± 2.8
Symptoms duration, months	44.5 ± 54.8
Previous knee surgery, yes/no	112/74
Concomitant knee surgery, yes/no	95/91
Kellgren-Lawrence grade	Grade 0: 38
	Grade 1: 102
	Grade 2: 35
	Grade 3: 11
	Grade 4: 0
Lesion size, cm^2^	3.3 ± 2.2
Lesion location	MFC: 71
	LFC: 43
	Patella: 42 Trochlea: 26 Tibial plateau:8
Number of lesions	Single: 182
	Double: 4
Etiology	Traumatic: 44
	Degenerative: 80
	OCD: 62

Values are expressed as mean ± standard deviation.

BMI = body mass index; M = men; MFC = medial femoral condyle; LFC = lateral femoral condyle; OCD = osteochondritis dissecans; W = women.

The procedure consisted in the implantation of a biomimetic scaffold (Maioregen, Finceramica Faenza Spa, Faenza, Italy) composed of a three-layer material that mimics the entire osteochondral anatomy.^21^ The scaffold was implanted by a team specialized in the treatment of cartilaginous lesions of the knee according to a previously described technique.^22^ Patients were evaluated through the IKDC subjective score and VAS for pain at baseline and at 12 and at 24 months after the cartilage treatment. Moreover, patients were asked at 12 and 24 months to express an overall opinion on the treatment received by answering an explicit anchor question, rating on a 6-point scale their clinical knee condition compared with the baseline: Compared with before the knee surgical treatment, how would you rate your knee now? (1, total recovery; 2, much better; 3, a little better; 4, no change; 5, a little worse; 6, much worse).

### MCID Quantification

The MCID was calculated using the distribution-based method derived from the value equal to half of the standard deviation of the overall cohort improvement for both IKDC subjective and VAS pain scores at 12 and 24 months of follow-up, as previously reported in the field literature.^18,23^ Patients were classified as reaching the MCID if it was achieved in > 1 of the outcome measures, as previously described.^18^ Moreover, to further investigate and confirm the obtained results with a more recently recommended methodological approach,^24,25^ the MCID was also calculated with the anchor-method as the mean observed score change in the “a little better” group for both IKDC subjective and VAS for pain scores at 12 and 24 months of follow-up.

### Statistical Analysis

All continuous data were expressed in terms of mean ± standard deviation, categorical variables were expressed as proportions or percentages. The Shapiro-Wilk test was performed to test normality of continuous variables. The Levene test was used to assess the homoscedasticity of the data. The Repeated Measures General Linear Model with Sidak test for multiple comparisons was performed to assess the differences of the scores between the basal and the follow-up times. The MCNemar non-parametric test was used to assess the differences in reaching the MCID between 12 and 24 months. The analysis of variance (ANOVA) test was performed to assess the difference between those who reached MCID and those who did not of continuous, normally distributed and homoscedastic variables, the Mann-Whitney test was used otherwise. The Spearman rank correlation was used to assess correlations between numerical scores and continuous data, the Kendall tau correlation was used to assess correlations between ordinal scores and continuous data. The Pearson chi-square evaluated using exact test was performed to investigate relationships between grouping variables and reaching the MCID. For all tests, *P* < 0.05 was considered significant. All statistical analyses were performed using SPSS v.19.0 (IBM Corp., Armonk, NY, USA).

## Results

**Figure 1.**
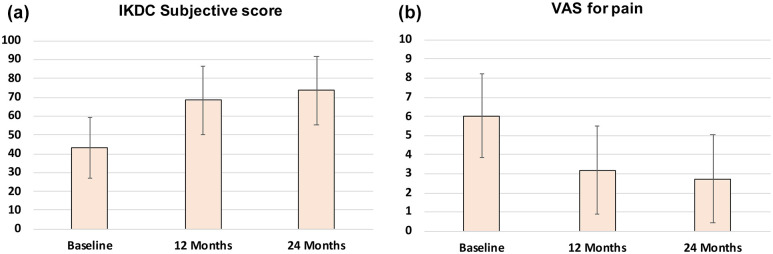
**(a)** International Knee Documentation Committee (IKDC) subjective score at baseline, 12, and 24 months. **(b)** Visual Analogue Scale (VAS) for pain at baseline, 12, and 24 months. Values are expressed as mean and standard deviation.

The IKDC subjective score and VAS for pain improved significantly from baseline to 12 months and from baseline to the 24-month follow-up (all *P* < 0.0005), as reported in Table 2. A further improvement was detected between 12 and 24 months for the IKDC subjective score (68.4 ± 18.5 vs. 73.7 ± 18.2; *P* < 0.0005) and for the VAS for pain score (3.2 ± 2.3 vs. 2.7 ± 2.3; *P* = 0.014). Figure 1 shows the trend of the two evaluated PROMs.

**Table 2.** IKDC Subjective Score and VAS for Pain Improvement From Baseline.

Score	Improvement at 12 months	Improvement at 24 months
IKDC subjective	25.1 ± 20.2	30.4 ± 20.2
VAS pain	2.8 ± 2.9	3.3 ± 3.1

IKDC = International Knee Documentation Committee; VAS = Visual Analogue Scale.

The MCID values for the IKDC subjective score calculated with the distribution-based method were 10.1 both at 12 months and at 24 months of follow-up. The MCID values for the VAS pain score calculated with the distribution-based method were 1.5 both at 12 and 24 months of follow-up. No differences between the values at 12 and 24 months were found for both PROMs. The rate of patients who achieved the MCID was 84% at 12 months and 88% at 24 months, without a significant increase of patients who achieved the MCID from 12 to 24 months of follow-up (*P* = 0.189).

Age and lesion site were found to influence the achievement of MCID thresholds at 24 months of follow-up. In detail, patients who reached the MCID at 24 months of follow-up were younger than patients who did not reach the MCID (31.2 ± 10.9 years vs. 35.8 ± 12.1 years, *P* = 0.042). Patients with lateral femoral condyle lesions were more likely to reach the MCID for the VAS pain score at 24 months compared with other lesion sites (86.0% vs. 77.8%; *P* = 0.002). Other demographic and clinical patients’ characteristics, such as sex, body mass index, symptoms duration, previous and concomitant knee surgeries, Kellgren-Lawrence grade, lesion size, number of lesions, and etiology did not influence the MCID calculated with the distribution-based method.

The anchor-based method was used as control and led to MCID values for the IKDC subjective score of 20.2 and 23.2 at 12 and 24 months of follow-up, respectively, and for the VAS for pain score of 2.4 and 2.6 at 12 and 24 months of follow-up, respectively. The rate of patients who achieved the MCID was 68% at 12 months and 75% at 24 months, with a significant increase of patients who achieved the MCID from 12 to 24 months of follow-up (*P* = 0.016). Sex and lesion site were found to influence the achievement of MCID thresholds. In detail, male patients were more likely to reach the MCID for VAS pain at 12 months compared with female patients (58.3% vs. 40.4%, respectively; *P* = 0.042). Patients with lateral femoral condyle lesions were more likely to reach the MCID compared with the other lesion sites for the IKDC subjective score at 24 months (79.1% vs. 62.2%; *P* =0.045) and for VAS pain at 12 (74.4% vs. 67.6%; *P* = 0.030) and 24 months (72.1% vs. 55.2%; *P* = 0.045). Moreover, patients with patellar lesions were less likely to reach the MCID compared with the other lesion sites for VAS pain at 12 months (37.5% vs. 59.0%; *P* = 0.009). Other demographic and clinical patients’ characteristics, such as age, body mass index, symptoms duration, previous and concomitant knee surgeries, Kellgren-Lawrence grade, lesion size, number of lesions, and etiology did not influence the MCID calculated with the anchor-based method.

## Discussion

The main finding of this study is the definition of MCID values for the IKDC subjective and VAS pain scores at 12 and 24 months postoperatively after treatment with a cell-free biomimetic scaffold for lesion of the knee articular surface. The MCID value of the IKDC subjective score was 10.1 at 12 and 24 months of follow-up, while the MCID value of the VAS for pain score was 1.5 at 12 and 24 months of follow-up. The identification of these thresholds can improve the understanding of the results of scaffold implantation in these patients, with a better interpretation of the clinical trials findings in terms of clinical relevance, while helping clinicians to counsel patients on the potential of scaffold implantation procedures.

The use of scaffold-based procedures to address knee chondral and osteochondral lesions is increasing in the orthopedic clinical practice.^7^ A growing number of clinical trials are exploring the clinical efficacy of these treatments, although specific MCID values are needed to determine the real clinical benefit perceived by patients. This study filled this gap in the literature by analyzing over 180 patients treated with a cell-free scaffold and followed prospectively up to 2 years, determining the MCID thresholds of for two PROMs commonly used for these lesions: IKDC subjective and VAS for pain scores. The definition of specific MCID values for a specific treatment is of clinical relevance, as it allows the application of these values rather than others for different treatments and thus less appropriate.^18-20^ In fact, it is known that MCID values can vary based on the considered treatment, even when similar populations were considered. In this light, the application of dedicated MCIDs is key to properly evaluate treatment outcomes.

This study defined the MCID values of the IKDC subjective score and the VAS for pain score at 12 and 24 months postoperatively, being 10.1 and 1.5, respectively. These values differ from those already calculated for the same PROM for other cartilage procedures, such as microfractures, autologous chondrocyte implantation, or osteochondral autografts, with IKDC subjective MCID values of 10, 16, and 17, respectively.^18-20^ Accordingly, the values of MCID for the IKDC subjective score of the different procedures showed a high variability in the literature, thus confirming that the type of treatment represents a key parameter that may contribute to the variation in the calculation and application of MCID thresholds for a given score. The application of specific MCID values helps reducing the risk of underestimating or overestimating the true clinical effectiveness of specific treatments for knee cartilage lesions, as well as to identify patient categories benefiting more or less from each procedure.

This study also evaluated the factors that can influence the MCID achievement of the patients after the scaffold implantation. Demographic patients’ characteristics, such as age and sex can influence the achievement of MCID. In particular, younger patients and males have higher possibility to achieve the MCID. These results reflect the previous reported findings on influencing factors on the clinical outcomes after scaffold implantation procedures, with age and sex showing to influence the clinical outcome.^26^ The lesion site does influence the MCID achievement after scaffold implantation, with patients suffering from lesions of the lateral femoral condyles reaching the MCID more frequently than other locations, such as the patella. The role of the lesion site on the MCID achievement is in line with previous literature. In a recent study by Bartha *et al*.,^27^ of 831 mosaicplasty procedures for condylar lesions, good results were obtained in 92% of cases while this rate was reduced to 79% for lesions of the patellofemoral joint. A similar difference has also been shown for autologous chondrocyte transplantation and matrix-assisted autologous chondrocyte transplantation.^28^ The explanation for this lower improvement for patellar lesions probably lies in the complex biomechanics of the patellofemoral joint, which is affected by high forces during flexion and multidirectional stresses due to the patellar and trochlear shape, muscle forces, stability, and alignment.^29^ Other demographic and clinical patients’ characteristics did not show to influence the MCID achievement in this cohort of patients over time.

The MCID-identified thresholds were stable showing the same values at 12 and 24 months for both PROMs evaluated. A different trend in MCID values over time was instead reported by Chahla *et al*.,^18^ who, using the same distribution-based method of the present study, established the MCID thresholds of the IKDC subjective score after microfractures in patients affected by knee cartilage lesions. These authors reported an MCID value at 12 months (10.3) comparable with that obtained in the current study, while they reported an increase in the MCID value (16.7) at 24 months. The authors suggested that this time-dependent variability of the MCID value could be explained by the high rate of failures after microfractures.^30^ To this regard, the stability over time of the MCID values in the cohort of patients treated with this scaffold could be related to the low number of treatment failures observed.^31^ The higher number of failures in the microfracture study introduces another important factor that may influence the MCID threshold definition, as a cohort with more failures may present a broader range of scores, which may affect the results obtained when applying the commonly used distribution-based approach.

The application of an anchor-based method, also used in the literature to calculate the MCID values, provided important insights on this matter, underlining how the use of different MCID calculation methods can lead to different values. In fact, the anchor-based method provided both higher thresholds and changes over time. This variability is explained by conceptual and methodological differences of the different calculation methods, each one with its own advantages and disadvantages.^12,32^ Distribution-based methods are purely statistical approaches that do not take into account the patients’ expectations through specific clinical questionnaires and are sample-specific, being strictly related to the characteristics of the clinical results of the population examined.^25,33^ On the contrary, the patients’ perspective is the key parameter of anchor-based methods, with over than dozens methods described, which estimate the MCID by reference to an external subjective assessment of the patient used to evaluate a change in a PROM.^34,35^ A previous analysis showed that the use of different calculation methods can lead to variability in the calculated MCID values.^14^ This heterogeneity can lead to problems of interpretation of the success of the treatment in a given population and can create a conflict in deciding which one of the reported MCID values is more appropriate.^17,36^ More light should be shed on the most suitable calculation method and future studies should better explore this aspect.

The current study has some limitations that should be considered when interpreting the results. Several patients underwent concomitant procedures and the heterogeneity introduced could have influenced the MCID assessment. However, since knee malalignment and ligament instability can be risk factors for cartilage procedure failures, concomitant procedures were performed to optimize the treatment success also in these patients by providing the required corrections. Moreover, the presence of concomitant procedures did not show to influence the achievement of the MCID value and a recent study on a large cohort of patients treated with this cell-free scaffold highlighted that concomitant procedures did not influence patient outcomes up to 2 years of follow-up.^26^ Furthermore, the sample size was in line with previous literature for the MCID assessment but may have hindered the possibility to perform subanalyses to detect factors able to influence the achievement of the MCID thresholds, subanalyses that were also affected by the heterogeneity among the different groups. Finally, as underlined by the two analyses performed in this study, an additional limitation could be the MCID calculation methodology itself, with only two MCID calculation methods used, while a broader number of alternative methods has been described in the literature. Each method has its own advantages and disadvantages, and a consistent superiority of one method over others has yet to be demonstrated. Future studies should explore the most suitable approach to calculate these thresholds to better understand patient perceptions of treatment outcomes. Nevertheless, the results of this study provide a reference for the field, both for surgeons and for patients. These findings offer surgeons some thresholds to better assess patient progress and are useful for the patients, suggesting predictive factors to consider to have proper expectations when undergoing cell-free scaffold implantation for the treatment of articular surface lesions.

## Conclusion

This study defined the MCID values for the IKDC subjective and VAS pain scores at 12 and 24 months postoperatively after treatment with a cell-free biomimetic scaffold, with 88% of the patients achieving clinically relevant results at 2 years. Not all patients benefited the same, with younger patients, males, and lateral femoral condyle lesions being more likely to reach the MCID. However, while these data can help understanding the clinical relevance of the treatment outcome, the identified thresholds can be influenced by the method chosen, which warrants caution when interpreting study results.
